# Nutritional Status and Associated Factors Among School Adolescent Girls in North Shoa Zone, Amhara Region, Ethiopia: A Cross-Sectional Study

**DOI:** 10.1089/whr.2022.0103

**Published:** 2023-03-27

**Authors:** Sisay Shine Tegegnework, Behailu Tariku Derseh, Wondoson Asegdew Meseret, Abayneh Birlie Zeru, Hilina Ketema Assefa, Awraris Hailu Bilchut, Sindew Mahmud Ahmed

**Affiliations:** ^1^ Public Health Department, Institute of Medicine and Health Science, Debre Berhan University, Debre Berhan, Ethiopia.; ^2^ Nursing Department, Institute of Medicine and Health Science, Debre Berhan University, Debre Berhan, Ethiopia.; ^3^ Nursing Department, Minlik II College of Medicine and Health Science, Kotebe Metropolitan University, Addis Ababa, Ethiopia.

**Keywords:** adolescent girls, nutritional status, stunting, thinness, Ethiopia

## Abstract

**Background:**

Adolescents are the most affected group and the group that is least studied when it comes to malnutrition, which is one of the most important public health concerns in underdeveloped countries, including Ethiopia. Therefore, the goal of this study was to assess nutritional status and related factors among teenage females who are in school.

**Materials and Methods:**

From October 1 to October 25, 2018, 645 young girls in school participated in an institution-based cross-sectional study. Girls in their teen years from the school were selected using a simple random sample method. Anthropometric tests and in-person interviews were used to collect the data. An odds ratio with 95% confidence intervals (CIs) and a *p*-value under 0.05 were used to declare a statistical association.

**Results:**

This study found that 12.3% and 9.6% of school adolescent girls were stunted and thin, respectively. Being a rural resident (adjusted odd ratio [AOR]: 1.85, 95% CI: 1.05–3.28) and inadequate dietary diversity score (AOR: 3.02, 95% CI: 1.06–4.60) were significant predictors of stunting. School adolescent girls from merchant father were 71% less likely to develop stunting compared with government employee father. Late adolescent age (AOR: 2.27, 95% CI: 1.28–4.02) and family size ≥5 (AOR: 1.98, 95% CI: 1.05–3.75) were significant predictors of thinness.

**Conclusions:**

Stunting and thinness were the major public health problems among school adolescent girls in the study area. Being a rural resident and inadequate dietary diversity increases the risk of stunting. However, being late adolescent girl and large family size were risk factors for thinness. There was a need for a school-based instruction program that concentrated on a varied diet and methods of encouraging the adolescent girls' appetites.

## Introduction

According to the World Health Organization (WHO), an adolescent is someone between the ages of 10 and 19 years.^1^ It is a crucial period in a person's life cycle that has a significant impact on their physical, psychological, and cognitive development. In this age group, adolescents gain 50% of their adult weight, more than 20% of their adult height, and 50% of their adult body mass.^2^ These variables make this age group's dietary needs greater than others throughout the human life span.^2,3^

In 2016, underweight affected more than 16.2 million adolescent girls globally. Particularly in sub-Saharan Africa, where up to 55% of adolescent females were underweight, the issue is serious in Asian and African nations. In addition to causing stunting and thinness in adolescents, poor nutrition throughout adolescence will have a generational impact on children born to women who were undernourished at that time. Adolescence is thus a crucial time to address issues such as poverty, gender discrimination, violence, poor nutrition, and health that are pervasive and are handed down from one generation to the next.^4,5^

In Ethiopia, children and adolescents made up more than 48% of the population, with adolescent girls making up 25%;^6^ however, this group is the most underserved. Ethiopia currently has a National Nutrition Program and national school health and feeding initiatives that were created by the government to improve adolescent nutrition.^7^ Studies conducted in a separate region of Ethiopia, however, revealed that dietary concerns continue to be one of the major public health problems affecting people at all stages of life,^8^ particularly among adolescent girls.

Stunting and thinness are prevalent in Ethiopian adolescent girls, with prevalence rates ranging from 6.8% to 33.2% and 8.82% to 58.3%, respectively.^9–12^ Numerous factors contribute to these conditions, including poor dietary diversity,^13^ adolescent residence,^14^ family size,^15^ household income,^16^ source of water,^17^ and availability of toilets at the household level.^9^ Information on nutritional status among school-aged adolescent girls is limited in the study area. Therefore, the purpose of this study was to evaluate the dietary variety, food frequency, nutritional status, and related characteristics among school-aged adolescent girls in the North Shewa Zone of Ethiopia's Amhara region.

## Materials and Methods

### Study setting

The Amhara regional state of Ethiopia's North Shoa Zone is where the study was carried out. Bahir Dar, the capital of the Amhara Regional State, is 695 km away, and Addis Abeba, the capital of Ethiopia, is 130 km away. The North Shoa Zone is expected to have a population of about 2,299,203 in 2019/2020, according to the data from the North Shoa Zone Plan Commission. Of these, 1,120,398 are women, making up 1.05 men for every 1 woman. Additionally, 85% of people reside in rural areas.^18^

### Study design and period

Community-based cross-sectional study design was conducted from October 1–30, 2018.

### Source and study population

The source population consisted entirely of adolescent girls who attended schools in the North Shoa Zone. The study population consisted of adolescent girls located in the North Shoa Zone's chosen schools. Adolescent girls in public schools who are in grades 5 and up and who are healthy during data collection met the study's inclusion criteria.

### Sample size determination

The sample size was established using a single population proportion formula under the following assumptions: 26.5% of Ethiopian school-aged girls who are stunted,^9^ a 95% confidence level of 1.96, and a 5% maximum permitted error. The final required sample size after using design effect of 2 and nonresponse rate of 10% was 658.

### Sampling procedures

The study participants were chosen using a multistage sampling technique. First, six districts were chosen at random from the zone's total of 24 districts. Second, three schools—one primary (5th–8th grade), one secondary (9th–10th grade), and one preparatory (11th–12th grade)—were chosen by lottery from each chosen district. The administrator of each institution provided a sample frame of adolescent girls before the actual data collection. Then, adolescent girls were chosen using basic random sampling methods.

### Data collection tools and methods

For the collection of quantitative data, a structured interview questionnaire that was modified from UNICEF and employed after evaluating various literatures of related research and anthropometric measures was used. Language experts originally created the questionnaire in English before translating it to Amharic and back to English to ensure uniformity. Six supervisors and 12 nurses were trained in data collecting. Adolescent females' weight was measured to the nearest 0.1 kg using a beam balance with the lamp closed, and their height was measured to the nearest 0.1 cm while they were standing without shoes. By initially asking the adolescent girl if she had eaten a certain type of food in the 24 hours before to the date of data collection, the categories for dietary diversity were established. These were subsequently merged to form several dietary categories. Adolescents' dietary diversity score (DDS) was evaluated, with scores of “poor” for food groups 0–3, “medium” for food groups 4–5, and “high” for food groups greater than 6.

### Operational definition

#### Adolescence

Adolescence is the age group from 10 to 19 years.^19^

#### Stunting

Stunting is defined as height-for-age below −2 Z-score of the 2007 WHO standard reference values.^20^

#### Moderate stunted

Moderate stunted is defined as height-for-age between −2 and −3 Z-scores of the 2007 WHO standard reference values.^20^

#### Severely stunted

Severely stunted is defined as adolescents whose height-for-age Z-score is below minus 3 (-3.0) standard deviations (SD) below the mean on the 2007 WHO Growth Standards.^20^

#### Thinness

Thinness is defined as body mass index (BMI) for age below −2 Z-score of the 2007 WHO standard reference values.^20^

#### Moderate thinness

Moderate thinness is defined as BMI for age between −2 and −3 Z-scores of the 2007 WHO reference values.^20^

#### Severely thinness

Severely thinness is defined as adolescents whose BMI for age Z-score is below minus 3 (-3.0) SD below the mean on the 2007 WHO Growth Standards.^20^

### Outcome variable

In this study, the outcome variable was nutritional status (stunting and thinness) of adolescent girls.

### Independent variables

Sociodemographic factors (age, place of residence, head of household, educational level of the mother and father, occupation of the mother and father, household monthly income, and family size) were the independent variables in this study. Other independent variables included appetite and environmental factors (changes in appetite for certain foods, presence of a functional toilet, source of drinking water, distance from source of water, and fasting), as well as dietary diversification (DDS) of adolescent girls.

### Data quality control

Interviewers, anthropometric recorders, and supervisors all received training on data gathering methodologies and procedures. Before the actual data collection, a pretest of the questionnaire was conducted. Anthropometric measurement scales were calibrated. The lead investigators and the supervisor went over the completed questionnaire each day to check for inaccuracies.

### Data processing and analysis

Data were coded, checked for accuracy, and entered into the EPI-DATA 3.1 software program. Adolescent girls' ages, heights, and weights were entered into the WHO Anthros plus version 1.0.4 software program to convert nutritional data into indices' Z-scores. The 1-day diversity questionnaire developed by the WHO and Food and Agriculture Organization was used to gauge dietary diversity. To evaluate the dietary diversity of school-aged adolescent girls, 14 food groups were used. For analysis, the entered data were exported to SPSS version 20.

To ascertain the relationship between each predictor and the result variables, bivariate logistic regression analysis was used. Then, variables from the bivariate analysis with 95% confidence intervals (CIs) and a *p*-value lower than 0.25 were included in the multivariate logistic regression analysis. Model fitness was assessed using the Hosmer–Lemeshow test, and multicollinearity among variables was assessed using the multicollinearity standard error. The related factors with thinness and stunting were identified using adjusted odds ratios with 95% CIs and *p*-values <0.05.

### Ethical approval and informed consent

After receiving ethical approval from the Department of Public Health Research Ethics Review Committee at the Institute of Medicine and Health Science at Debre Berhan University (Ref. No./IOMHS/028/201/10/2018), this study was carried out. Following receipt of an ethical clearance notice from the Zone administrative health bureau, data collecting was started. Each study participant's parents provided written consent before any data were collected. Each participant's parents read the letter, which was then further clarified by the data collectors so that everyone could comprehend the study's goal. The consent form was then signed by the parents of each participant.

## Results

### Sociodemographic characteristics of the study participants

The study included 627 school-aged adolescent girls in total, with a response rate of 98.6%. Three hundred seventy-nine participants in the study, or more than half (60.4%), fell into the age range of 10–15 years. The mean age of school-aged adolescent girls was 14.92 years, with a SD of +2.04. More than half (54.1%) of the total study participants were in elementary school and 53.3% from a rural location. The majority of respondents, 597 (95.2%) and 624 (99.5%), were Amharas and Ethiopian Orthodox Christians, respectively. The majority of adolescent school girls' mothers (59.3%) and fathers (73.0%) were housewives and farmers, respectively. More than one third (36.2%) of adolescents' mother educational status were cannot read and write, and half of their father can read and write (50.2%) (Table 1).

**Table 1.** Sociodemographic Characteristics of Study Participants in North Shoa Zone, Amhara Region, Ethiopia 2018

Variable	Frequency	%
Age (in years) category		
10–14	247	39.4
15–19	380	60.6
Residence		
Rural	334	53.3
Urban	293	46.7
Religion		
Orthodox	597	95.2
Muslim	19	3.0
Protestant	11	1.8
Ethnicity		
Amhara	624	99.5
Other	3	0.5
Head of the household		
Father	480	76.6
Mother	147	23.4
Educational status of mother		
Cannot read and write	227	36.2
Can read and write	240	38.3
1st–8th grade	109	17.4
9th–12th grade	26	4.1
Certificate and above	25	4.0
Educational status of father		
Cannot read and write	111	17.7
Can read and write	315	50.2
1st–8th grade	118	18.8
9th–12th grade	48	7.7
Certificate and above	35	5.6
Occupation of the mother		
Housewife	372	59.3
Merchant	127	20.3
Government employee	128	20.4
Occupation of the father		
Farmer	458	73.0
Merchant	94	15.0
Government employee	75	12.0
Family size		
<5	382	60.9
≥5	245	39.1

### Dietary diversification among school adolescent girls

**FIG. 1.**
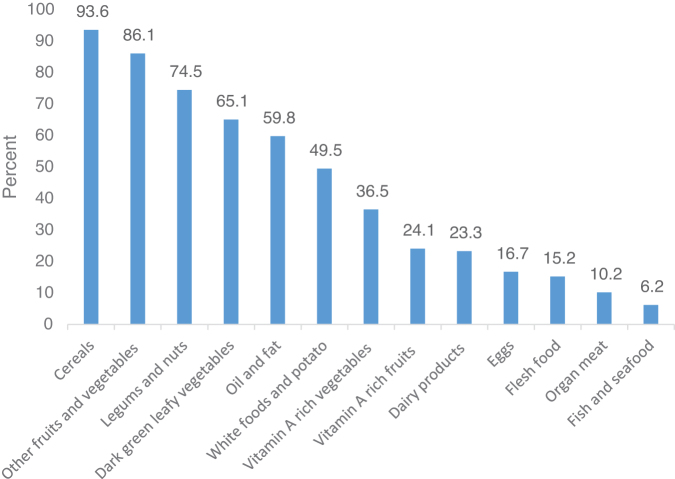
Type of food groups consumed in 24 hours by school adolescent girls in North Shoa Zone, Amhara region, Ethiopia 2018.

Girls in high school had a mean DDS of 5.6 and an SD of ±2.2. More than half (52.6%) of the study subjects practiced less dietary diversity than the average. Within a 24-hour period, 35.2% of the study's participants reported eating a meal away from home. The majority of adolescent girls (46.3%) ingested more than or equal to six food groups, whereas just 15.3% consumed three food groups or fewer. Cereals were ingested by a sizable portion of adolescent girls (93.6%), followed by other fruits and vegetables (86.1%). But only 6.2% of people consumed fish and seafood (Fig. 1).

### Meal consumption pattern among school adolescent girls

**FIG. 2.**
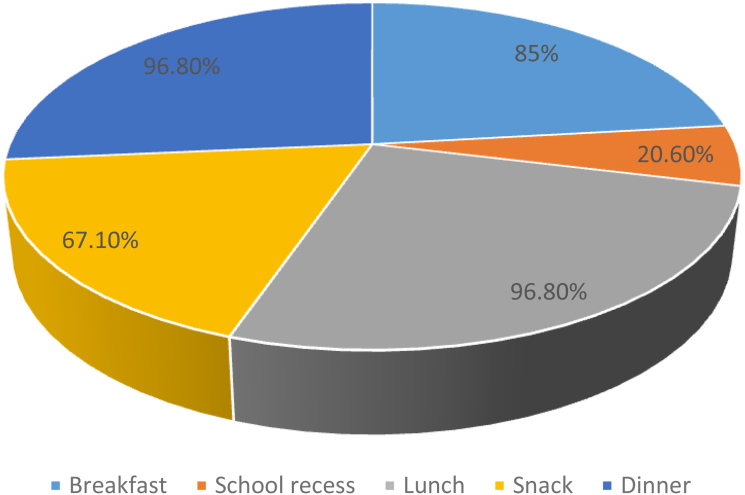
Meal consumption pattern of school adolescent girls in North Shoa Zone, Amhara region, Ethiopia 2018.

The majority (93.6%) of the study's total participants ate more than three meals every day. The majority of the school's adolescent girls (96.8%) took their main daily meals, which were lunch and dinner. Only 20.6% of the adolescents at the school took a snack, compared with more than 85% of the adolescent girls who ate breakfast (Fig. 2).

In the 7 days before the study, 68.8% of the school-aged girls had never had milk or milk products. Of the total study participants, 23.3% ingested grain products regularly and 41.2% occasionally. In the 7-day recall, 55.7% of school-age girls reported drinking water every day, whereas 48.3% reported drinking tea or coffee daily (Table 2).

**Table 2.** Food Consumption Pattern of School Adolescent Girls in North Shoa Zone, Amhara Region, Ethiopia 2018

Food group	Frequency	%
Milk, yogurt, or soymilk, cheese		
Daily	12	1.9
More than three daily	33	5.3
A few times a week	151	24.1
Rarely or never	431	68.7
Vegetables		
Daily	97	15.5
More than once daily	93	14.8
A few times a week	316	50.4
Rarely or never	121	19.5
Fruits		
Daily	85	13.6
More than once daily	74	11.8
A few times a week	298	47.5
Rarely or never	170	27.1
100% Fruit juice		
Daily	18	2.9
More than once daily	32	5.1
A few times a week	167	26.6
Rarely or never	410	65.4
Grains/breads, injera, noodles, rice, pasta		
Daily	146	23.3
More than one daily	96	14.7
A few times a week	262	41.2
Rarely or never	127	20.3
Meat/poultry/fish/beans/eggs/nuts/seeds		
Daily	58	9.3
More than once daily	41	6.5
A few times a week	201	32.1
Rarely or never	327	52.2
Tea/coffee		
Daily	303	48.3
More than once daily	162	25.8
A few times a week	97	15.5
Rarely or never	65	10.4
Water		
Daily	349	55.7
More than once daily	218	34.8
A few times a week	28	4.5
Rarely or never	32	5.1
Fruit-flavored drinks, soft drinks		
Daily	58	9.3
More than once daily	36	5.7
A few times a week	245	39.1
Rarely or never	288	45.9

### Appetite and environmental health characteristics of school adolescent girls

According to the study, 46.6% of school-aged adolescent females describe their appetite as very good, and 39.6% of them report having changed appetites for particular items. Seventy-one percent of the study participants were fasting when the data were being gathered. Most survey participants (49.7%) used public piped water as their primary source of domestic water. To get water for their homes, 74.0% of school-aged teenage females had to walk for an average of 20.8 minutes, with a SD of +10.6 minutes. Of these, 55.8% took longer. Nearly 90% of research participants had a functioning toilet in their home (Table 3).

**Table 3.** Appetite and Environmental Health Characteristics of School Adolescent Girls in North Shoa Zone, Amhara Region, Ethiopia 2018

Variable	Frequency	%
Had appetite change		
Yes	248	39.6
No	379	60.4
Appetite rate of the participants		
Very good	292	46.6
Good	172	27.4
Fair	138	22.0
Poor	25	4.0
Girls at fasting at time of data collection		
Yes	450	71.8
No	177	28.2
Household source of drinking water		
Private piped water	238	38.0
Public piped water	312	49.7
Protected well	27	4.3
Unprotected well	15	2.4
Spring	35	5.6
Girls fetch water for households		
Yes	464	74.0
No	163	26.0
Average time took to fetch water (*n* = 464)		
≤20 minutes	205	44.2
>20 minutes	259	55.8
Availability of functional toilet at household		
Yes	564	90.0
No	63	10.0

### Prevalence of stunting and thinness among adolescent girls

**FIG. 3.**
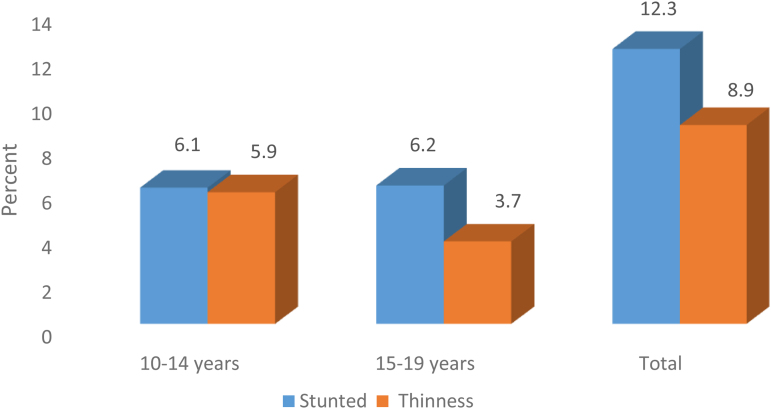
Prevalence of stunting and thinness among adolescent girls with their age category in North Shoa Zone, Amhara region, Ethiopia 2018.

In general, 12.3% (95% CI: 9.7–14.8) of adolescent girls were stunted, of which 11.0% (95% CI: 8.6–13.6) were moderately stunted and 1.3% (95% CI: 0.5–2.2) were severely stunted, according to the WHO classification. Adolescent girls had a 9.6% (95% CI: 7.2–11.8) prevalence of being thin overall, with 8.9% (95% CI: 6.9–11.2) being moderately thin and 0.6% (95% CI: 0.2–1.4) being very thin. Adolescent girls in primary schools were 7.7% and 7.2% more likely to be stunted and thin, respectively. Adolescent girls between the ages of 10 and 14 years were more likely to be thin (5.9%) (Fig. 3).

### Factors associated with stunting

Adolescent girls who lived in rural areas were 1.85 times (adjusted odd ratio [AOR]: 1.85, 95% CI: 1.05–3.28) more likely than urban dwellers to have stunting. When compared with their contemporaries, school-age girls whose mothers were the head of the home had a 2.12 times (AOR: 2.12, 95% CI: 1.06–4.23) higher risk of developing stunting. Adolescent school girls with low DDSs exhibited stunting rates that were 2.21 times higher than those with high DDSs (AOR: 2.21, 95% CI: 1.06–4.60). School-aged children whose father worked as a merchant had a 71% lower risk of stunting (AOR = 0.29, 95% CI: 0.10–0.87) than children whose father worked in government (Table 4).

**Table 4.** Factors Associated with Stunting Among School Adolescent Girls in North Shoa Zone, Amhara Region, Ethiopia 2018

Variable	Stunting		COR (95% CI)	AOR (95% CI)
	Yes (%)	No (%)		
Age (in years)				
10–14	38	209	1.59 (0.39–2.02)	0.65 (0.40–1.07)
15–19	39	341	1.00	1.00
Residence				
Rural	49	285	1.63 (1.01–2.67)	1.85 (1.05–3.28)^*^
Urban	28	265	1.00	1.00
Head of the household				
Father	50	430	1.00	1.00
Mother	27	120	1.94 (1.01–3.84)	2.12 (1.06–4.23)^*^
Occupation of the father				
Farmer	57	401	1.99 (0.20–2.30)	0.63 (0.23–1.68)
Merchant	15	79	2.66 (0.13–3.09)	0.29 (0.10–0.87)^*^
Government employee	5	70	1.00	1.00
Dietary diversification				
Inadequate DDS	28	103	1.94 (1.08–4.58)	2.21 (1.06–4.60)^*^
Moderate DDS	15	204	0.52 (0.88–2.55)	1.66 (0.95–2.88)
Adequate DDS	34	243	1.00	1.00
Girls fetch water for households				
Yes	63	401	1.67 (0.33–2.10)	0.64 (0.35–1.20)
No	14	149	1.00	1.00

^*^Significant at *p* < 0.05.

AOR, adjusted odd ratio; COR, crud odd ratio; DDS, dietary diversity score.

### Factors associated with thinness

Adolescent girls in late school had a 2.27 times (AOR: 2.27, 95% CI: 1.28–4.02) higher likelihood of being skinny than their peers. Adolescent girls in school who came from a family of five or more were 1.98 times (AOR: 1.98, 95% CI: 1.05, 3.75) more likely to be thin than those who came from a family of five or fewer (Table 5).

**Table 5.** Factors Associated with Thinness Among School Adolescent Girls in North Shoa Zone, Amhara Region, Ethiopia 2018

Variable	Thinness		COR (95% CI)	AOR (95% CI)
	Yes	No		
Age (in years)				
10–14	23	357	1.00	1.00
15–19	37	210	2.74 (1.58–4.73)	2.27 (1.28–4.02)^**^
Occupation of the mother				
Housewife	40	332	1.00	1.00
Merchant	8	119	0.56 (0.40–3.94)	1.68 (0.75–3.75)
Government employee	12	116	0.86 (0.59–2.30)	1.31 (0.65–2.64)
Family size				
<5	46	336	1.00	1.00
≥5	14	231	0.44 (0.87–2.82)	1.98 (1.05–3.75)^*^
Had appetite change				
Yes	18	230	0.62 (0.89–2.84)	1.48 (1.08–2.7)^*^
No	42	337	1.00	1.00
Appetite rate of the participants				
Very good	33	259	1.00	1.00
Good	10	162	0.48 (0.18–1.45)	1.51 (0.71–3.22)
Fair	12	126	0.74 (0.67–2.68)	1.04 (0.51–2.14)
Poor	5	20	1.96 (0.99–4.30)	0.41 (0.13–1.24)

^*^ Significant at *p* < 0.05.

^**^ Significant at *p* < 0.001.

## Discussion

Poor nutrition among adolescent girls in schools continues to be a serious public health issue in developing nations such as Ethiopia. This study found that among adolescent girls, the prevalence of stunting was 12.3% (95% CI: 9.7–14.8). It exceeded the 6.8% observed in the Addis Ababa, Ethiopia research.^10^ In contrast, it was lower (34.0%) compared with the research conducted in Bangladesh.^21^ Indonesia (25.0%),^22^ rural India (19.2%),^23^ West Bengal in India (20.7%) and (31.4%),^24,25^ and various regions of Ethiopia^9,10,12–16,26–29^ are among the countries with the highest percentages. These disparities could have a long-term nutritional influence on school-aged adolescent girls due to seasonal variance, economic condition, and educational attainment of the families.

According to the study's results, school-aged teenage girls made up 9.6% (95% CI: 7.2–11.8) of the population. It was in line with studies conducted in Ethiopia (8.82%),^27^ Iran (10.1%),^30^ and Ghana (10%).^31^ Nevertheless, it was higher than the research reported in Indonesia (5.0%)^22^ and Ethiopia (1.4%).^10,15^ This study, in contrast, came in lower than the research reported in India (28.2%)^23^ and Ethiopia.^9,10,13,16,26,32–35^ This discrepancy could be attributed to the different data collection periods, dietary habits of the community (such as the fact that skipping breaks [15%] was common in the study area), and the inclusion of multiple schools in the current study, which can be found in both rural and urban areas, as opposed to most other studies, which focus on a single school in either an urban or rural community and may have an impact on the prevalence of thinness.

According to this study, adolescent girls who lived in rural areas had 1.85 times higher risks of developing stunting than those who lived in urban areas (AOR: 1.85, 95% CI: 1.05–3.28). It agreed with the research conducted in various parts of Ethiopia.^14,36^ This may be caused by the fact that rural inhabitants lack access to clean water and sanitary facilities, which can lead to diarrheal infections in adolescent females on several occasions. Stunting may also result from differences in the quantity and quality of food available to locals.

Mothers who are the head of the family are 2.12 times (AOR: 2.12, 95% CI: 1.06–4.23) more likely than their counterparts to experience stunting. It did not line up with the research performed in the Ethiopian towns of Chiro and Wolayta Sodo.^8,37^ This disparity may be the result of the different research populations used in the two urban studies, where the gender of the household head may not make a meaningful difference. However, men frequently serve as their homes' primary economic resource providers and decision-makers in Ethiopia's rural areas. But additional long-term research is required for this component. It is therefore possible to research how household heads affect the nutritional status of adolescent girls.

Similar to this, adolescent girls were 2.21 times (AOR: 2.21, 95% CI: 1.06–4.60) more likely to experience stunting than those with adequate dietary diversification scores. It agreed with the research conducted in various parts of Ethiopia.^13,27,29,32,34^ This might be the case because dietary diversity is a proxy indicator of dietary habits and has the capacity to record consumption of both macronutrients and micronutrients. Studies conducted in other regions of Ethiopia, however, revealed no connection between DDS and stunting in adolescent girls.^26^

In this study, late adolescent girls had 2.27 times the odds of being thin compared with early adolescent girls (AOR: 2.27, 95% CI: 1.28–4.02). It did not line up with the research performed in other parts of Ethiopia.^9,10,13,32,34^ This discrepancy may be caused by the late onset of menarche, which can cause acute nutritional depletion in adolescents, and the late age at which the study area's adolescents had experienced fasting for religious purposes.

School-aged adolescents from families with five or more members had 1.98 times the odds of being thin compared with families with fewer members (AOR: 1.98, 95% CI: 1.05, 3.75). It was in line with the research performed in India,^23^ Saudi Arabia,^38^ and Ethiopia.^10,12,15,17,36^ This may be the result of a large family sharing the available food, which results in insufficient food consumption and thinness. However, a different Ethiopian study found no connection between family size and thinness.^33^

We should be aware of the limitations in this study. The use of cross-sectional studies may make it difficult to establish a true causal link between school-aged girls' nutritional status (stunting and thinness) and its risk factors. We only include adolescent girls who attend public schools and leave out those who attend private institutions. This makes it potentially representative of all adolescent girls in schools.

## Conclusions

Stunting and thinness were found to be significant public health issues in the research area. Significant risk factors for stunting included place of residence, home headship, father's profession, and dietary variety. Among school-aged adolescent girls, being slim was substantially correlated with age, family size, and changes in food preferences. The majority of adolescent girls in school have varied eating habits. Almost one fourth of school-aged adolescent girls eat their snack, whereas one in six adolescent girls has skipped breakfast. Therefore, it is advised that nutrition intervention programs be expanded with an emphasis on increasing dietary diversity among school-age girls, paying particular attention to rural schoolgirls.

## Abbreviations Used

ANC: antenatal care
AOR: adjusted odd ratio
BMI: body mass index
CI: confidence interval
COR: crud odd ratio
DDS: dietary diversity score
NNP: National Nutritional Program
SD: standard deviation
WHO: World Health Organization
